# The chromosome-level genome assembly and genes involved in biosynthesis of nervonic acid of *Malania oleifera*

**DOI:** 10.1038/s41597-023-02218-8

**Published:** 2023-05-19

**Authors:** Tianquan Yang, Rengang Zhang, Xiaoling Tian, Gang Yao, Yuanting Shen, Sihai Wang, Jianfeng Mao, Guangyuan Li, Aizhong Liu, Weibang Sun, Yongpeng Ma

**Affiliations:** 1 Yunnan Key Laboratory for Integrative Conservation of Plant Species with Extremely Small Populations/Germplasm Bank of Wild Species, Kunming Institute of Botany, Chinese Academy of Sciences, Kunming, 650201 Yunnan China; 2 University of the Chinese Academy of Sciences, Beijing, 100049 China; 3 Institute of International Rivers and Eco-Security, Yunnan University, Kunming, 650500 China; 4 Yunnan Academy of Forestry, Kunming, 650201 China; 5 Department of Plant Physiology, Umeå Plant Science Centre, Umeå University, Umeå, SE-901 87 Sweden; 6 Beijing Ori-Gene Science and Technology Co. Ltd, Beijing, 102206 China; 7 Key Laboratory for Forest Resource Conservation and Utilization in the Southwest Mountains of China, Southwest Forestry University, Kunming, 650224 China

**Keywords:** Genomics, Genome

## Abstract

Nervonic acid (C24:1 Δ15, NA) is a very long-chain monounsaturated fatty acid, a clinically indispensable resource in maintaining the brain and nerve cells development and regeneration. Till now, NA has been found in 38 plant species, among which the garlic-fruit tree (*Malania oleifera*) has been evaluated to be the best candidate for NA production. Here, we generated a high-quality chromosome-scale assembly of *M. oleifera* employing PacBio long-read, short-read Illumina as well as Hi-C sequencing data. The genome assembly consisted of 1.5 Gb with a contig N50 of ~4.9 Mb and a scaffold N50 of ~112.6 Mb. ~98.2% of the assembly was anchored into 13 pseudo-chromosomes. It contains ~**1123 Mb** repeat sequences, and 27,638 protein-coding genes, 568 tRNAs, 230 rRNAs and 352 other non-coding RNAs. Additionally, we documented candidate genes involved in NA biosynthesis including 20 *KCSs*, 4 *KCRs*, 1 *HCD* and 1 *ECR*, and profiled the expression patterns of these genes in developing seeds. The high-quality assembly of the genome provides insights into the genome evolution of the *M. oleifera* genome and candidate genes involved in NA biosynthesis in the seeds of this important woody tree.

## Background & Summary

**Fig. 1:**
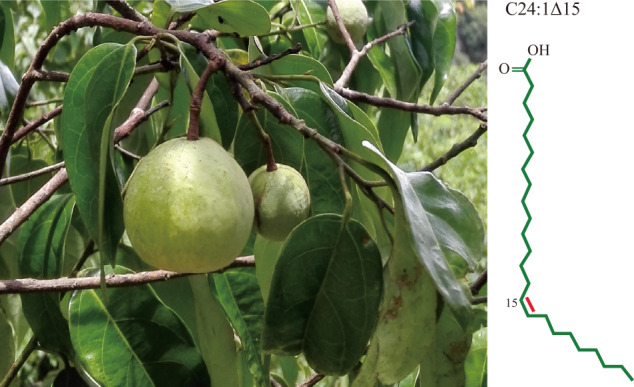
*M. oleifera* fruits with a representation of the chemical structure of nervonic acid.

Nervonic acid (C24:1 Δ15, NA) is a very long-chain monounsaturated fatty acid, originally isolated in shark brain tissues about 100 years ago^1^ (Fig. 1). As essential component comprising the white matter and myelin sheath of nerve fibers by combining with sphingosines to form nervonyl sphingolipids, NA has been found to be clinically indispensable and critical in maintaining the brain and nerve cells development and promoting the repair and regeneration of nerve fibers in damaged brain tissues^2^. With limited availability of marine creatures, NA derived from vegetable oils are alternative resources for supply of pharmaceutical and nutraceutical applications. Till now, NA has been found in 38 plant species belonging to 31 genera and 13 families, among which *Malania oleifera* has been evaluated to be the best candidate for NA production as *M. oleifera* has the highest content of NA reported thus far in any seed fat^2^ (Fig. 1). The *M. oleifera* genome has been sequenced and is publicly available^3^, but this assembly is fragmented and does not reach the chromosomal scale, which is impeding genome evolution analysis and genes’ identification and characterization. In the present study, the high-quality chromosome-scale assembly of the *M. oleifera* genome is provided. Then we further documented key genes involved in the NA biosynthesis by integrating the high quality genome information and transcriptome data.

**Fig. 2:**
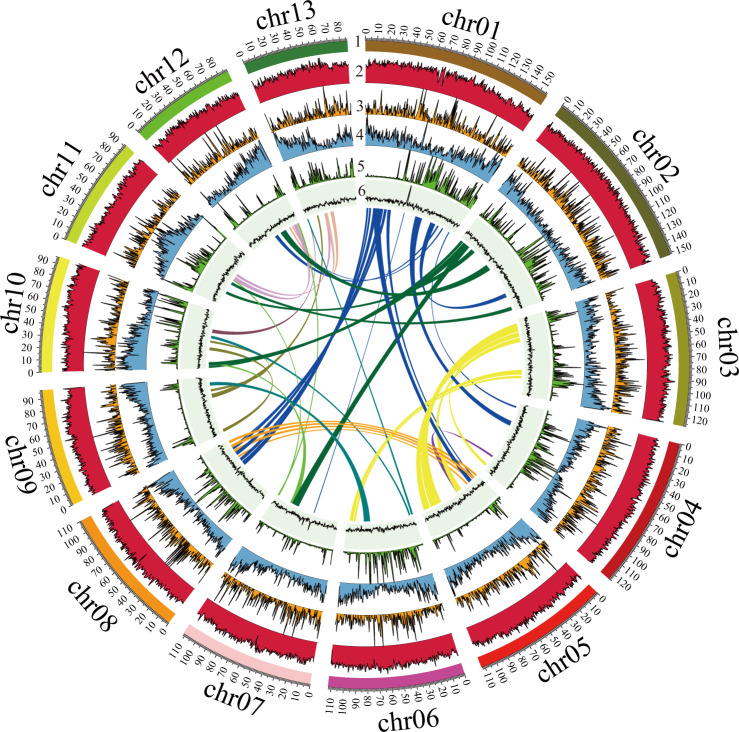
Circular map of *M. oleifera*. (1) Density of class I transposable elements, (2) Density of class II transposable elements, (3) Density of protein-coding genes, (4) Density of single nucleotide polymorphisms, (5) GC content, (6) Paralog synteny relationships.

**Fig. 3:**
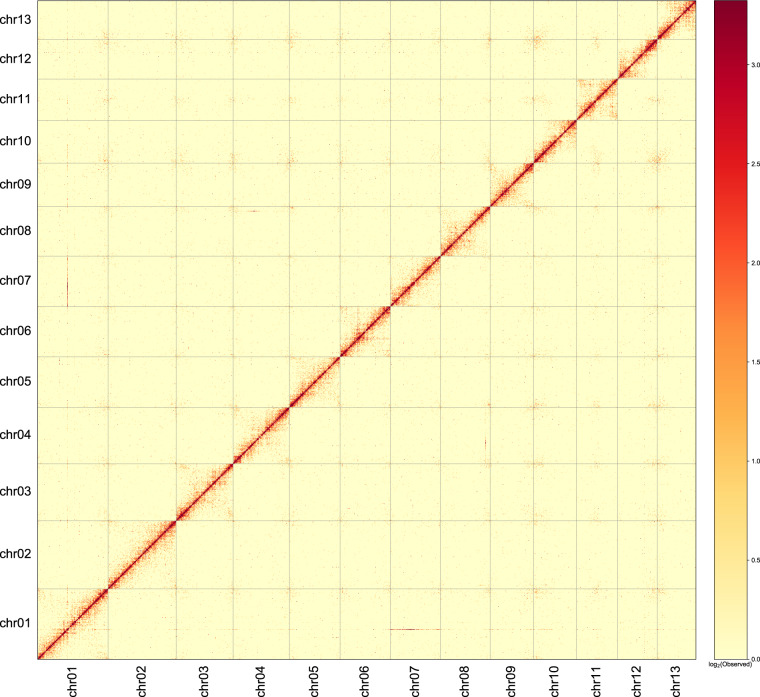
The Hi-C heatmap of chromosome interactions in *M. oleifera* chromosomes.

**Fig. 4:**
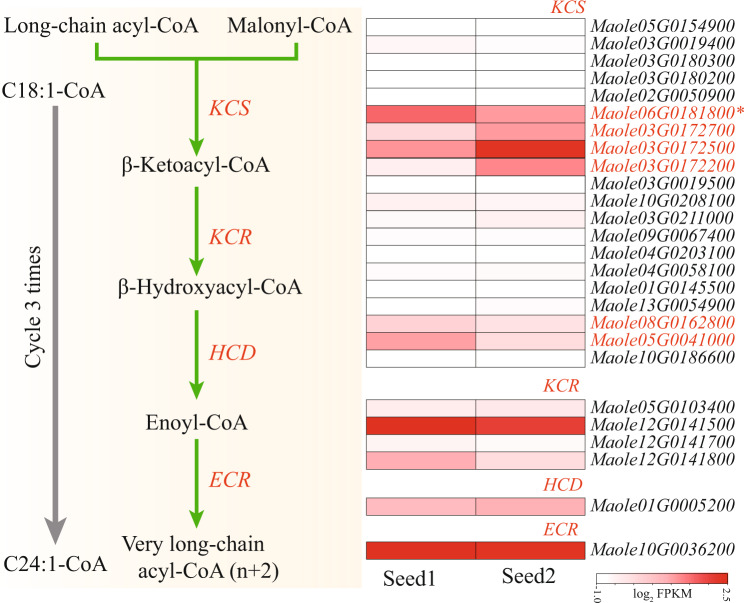
The biosynthetic pathway of very long-chain fatty acids including nervonic acid (C24:1, NA) and the expression of key genes in developing seeds (seed1: the initial stage of seed development with 0.88% NA, and seed2: the fast oil accumulation stage with 29.4% NA).

Based on an integrated approach, including PacBio long-read sequencing (51.1 Gb) and short-read Illumina sequencing (135.0 Gb) as well as Hi-C sequencing (173.0 Gb), a chromosome-scale assembly for the *M. oleifera* genome has been generated. The final *M. oleifera* genome assembly consisted of 1.5 Gb with a contig N50 of ~4.9 Mb and a scaffold N50 of ~112.6 Mb (Table 1). 98.2% (1468.2 Mb) of the assembly was anchored into 13 pseudo-chromosomes (Figs. 2, 3). The continuity of this genome assembly is significantly higher than that of the previous version, with contig N50 being 4 times higher and scaffold N50 being 24 times higher, and with a significant reduction in gap numbers (596 v.s. 1710; Table 1). Of the assembled genome sequence, 75.1% (1123.1 Mb) were transposable elements with a dominance of long terminal repeats (LTRs), which accounted for 68.0%. The most abundant repeat element families were Copia (28.9%) and Gypsy (30.0%) (Fig. 2, Table 2**)**. We also annotated 27,638 protein-coding genes (33,130 transcripts), 568 tRNAs, 230 rRNAs and 352 other non-coding RNAs (Tables 1, 3). The percentage of complete BUSCOs of genes was 92.2%, with 4.0% missing BUSCOs. The high-quality assembly provides opportunities for documenting key genes involved in the biosynthesis of very long-chain fatty acids including NA (Fig. 4). In total, we documented 20 genes encoding 3-ketoacyl-CoA synthase (KCS), four encoding 3-ketoacyl-CoA reductase (KCR), one encoding 3-hydroxacyl-CoA dehydratase (HCD) and one encoding trans-2, 3-enoyl-CoA reductase (ECR) (Fig. 4). Based on our previous transcriptome data generated from the two stages of *M. oleifera* seed development^4^, we revealed that six *KCS*s, all *KCRs*, *HCD* and *ECR* were expressed in the seeds with a FPKM value > 2 (Fig. 4), suggesting that these key genes were most likely responsible for NA biosynthesis. In conclusion, this high-quality assembly of the *M. oleifera* genome provides new insights into the evolution of the *M. oleifera* genome and valuable resources for metabolic engineering for the production of NA in other crops lacking NA.

**Table 1:** Comparison of original and updated *M. oleifera* genome assemblies and annotations.

Items	Genome sketch*	This study
Assembled genome size (Mb)	1519.8	1495.1
Number of scaffolds	1277	514
Scaffold N50 (Mb)	4.6	112.6
Number of contigs	2987	1110
Contig N50 (Mb)	1.2	4.9
Number of gaps	1710	596
Number of protein-coding genes	24,064	27,638
Repeat content (%)	82	75
Average CDS length (bp)	1281.1	1187.7
Average exon number per gene	6.0	5.7
Average exon length (bp)	244.5	261.4
GC content (%)	35.8	36.1

*Genome sketch is the previous published assembly from Xu *et al*.^3^.

**Table 2:** Comparison of genome repeat annotation of *M. oleifera*.

Items	Genome sketch	This study
LTR (%)	58.2	68.1
Copia (LTR)	29.5	28.9
Gypsy (LTR)	28.2	30.0
LINE (%)	3.7	1.5
SINE (%)	0.1	0.0
TIR (%)	3.3	1.3
Helitron (%)	0.1	0.0
Unknown (%)	11.9	1.5

**Table 3:** Statistics of gene functional annotations of *M. oleifera*.

Items	Swiss_Prot	TrEMBL	NR	*A. thaliana*
Gene numbers	16114	22764	22517	19304
Percentage	58.3%	82.4%	81.5%	69.9%

## Methods

### Sample collection and sequencing

Materials for high-throughput chromosome conformation capture sequencing were taken from a young tree of *M. oleifera*. The Hi-C library was prepared by Beijing Ori-Gene Science and Technology Co., Ltd (Beijing, China). A total of 700 ng of high molecular weight genomic DNA was cross-linked *in situ*, extracted, and subsequently digested with a restriction enzyme. The sticky ends of the digested fragments were biotinylated, enriched and sheared to a fragment size of 300–500 bp for preparing the sequencing library. Sequences were obtained from HiSeq X Ten platform (Illumina).

### Chromosome-level genome assembly

We started the genome assembly from the previous scaffold-level assembly of *M. oleifera*^3^. Gaps of the genome assembly were filled with third generation data using LR_Gapcloser v. 3^5^, run for two rounds to enhance contig continuity. Then pilon v. 1.24 program^6^ was used to polish one round with second generation data to enhance base-level accuracy. Subsequently the scaffolds were broken into contigs for downstream assembly. For Hi-C data-based assembly, Hi-C data were first mapped to the preprocessed genome using Juicer v. 0.6.8^7^, followed by preliminary Hi-C-assisted chromosome assembly using 3d-DNA v. 180922^8^, and then the Juicebox module^9^ for manual adjustment of chromosome boundaries. Then all chromosomes were re-scaffolded one by one with 3d-DNA, and then manually adjusted to correct errors with Juicebox module, including adjusting boundaries, removing wrong insertions and adjusting orientation. LR_Gapcloser v.3 and NextPolish v. 1.3.1^10^ were used for the final optimization. Redundans v. 0.14a^11^ was used to remove redundancy in un-anchored sequences (identity >= 0.98). We evaluated the step of redundancy removal by mapping Pabio reads (minimap2 v. 2.24 -x map-pb) to the assembly prior to redundancy removal.

### The quality and completeness evaluation of the assembled genome

We performed the following four assessments of the new genome: (i) completeness assessment; (ii) redundancy assessment; (iii) assessment of base-level accuracy and heterozygosity rate; and (iv) evaluation of Hi-C interaction. LTR Assembly Index (LAI)^12^ was examined using LTR_retriever v. 2.9.0^13^. We also assessed the genome using BUSCO v. 5.3.2^14^ with the embryophyta_odb10 lineage dataset for the proportion of intact core genes (both single-copy and multi-copy) and the proportion of missing genes. In the redundancy and heterozygosity assessment, KAT v. 2.4.2^15^ was used by comparing the genome and the corrected Pacbio reads. For the assessment of base-level accuracy and completeness, Merqury v. 1.3 was applied^16^.

### The annotation of the genome

RepeatModeler v. 2.0.1^17^ was employed to generate a repeat sequence library. The intact LTR-RTs were identified using LTR_retriever v. 2.9.0^13^ and clustered to generate another library. Two libraries were merged and repeat regions on the genome were identified using RepeatMasker v. 2.1^18^.

EST and homologous protein evidence for gene annotation were directly used for the 57,298 transcript sequences and 135,881 protein sequences obtained from the genome sketch^3^. For *ab initio* gene prediction, the parametric model of AUGUSTUS v. 3.4.0^19^ was trained with single-copy core genes identified by BUSCO v. 3.0.2^14^ and optimized for five rounds. MAKER2 v. 2.31.9^20^ annotation process was first run for one round; then the high quality gene set with Annotation Edit Distance (AED) score less than 0.1 was selected from the annotation results and trained again for AUGUSTUS v. 3.4.0^19^ and perform 5 rounds of optimization, followed by the final round of annotation by MAKER2 v. 2.31.9^20^ without AED selection. Through manual check, we found that there were many false fission genes from MAKER2. Thus, we corrected these issues with gene annotations of the PASA v. 2.4.1^21^ pipeline based on transcript evidence. The completeness of gene annotation was evaluated with BUSCO v. 5.3.2^14^. In addition, tRNA, rRNA and other non-coding RNAs were identified by using tRNAScan-SE^22^, barrnap (https://github.com/tseemann/barrnap) and RfamScan^23^, respectively.

The functions of protein-coding genes were annotated based on three strategies: (i) eggNOG-mapper v. 2.0.1^24^ annotation, which annotates the functions of genes by comparing with the eggNOG v. 5.0 homologous gene database, including Gene Ontology (GO) and Kyoto Encyclopedia of Genes and Genomes (KEGG) database. (ii) To identify the best comparison of protein domains, sequence similarity search in which protein sequences were compared with protein databases including Swiss_Prot (https://www.sib.swiss/swiss-prot), TrEMBL (https://www.uniprot.org/statistics/TrEMBL), NR (https://www.ncbi.nlm.nih.gov/refseq/about/nonredundantproteins) and Arabidopsis protein database (https://www.arabidopsis.org/portals/proteome/proteinLocation.jsp) was conducted using diamond v. 2.0.4^25^. The comparison criteria were set >30% and E-value < 1e-5. (iii) Structural domain similarity search: the sub-databases PRINTS, Pfam, SMART, PANTHER, CDD in the InterPro database (www.ebi.ac.uk/interpro) were compared using InterProScan v. 82.0^26^ to obtain the protein information.

### Identification and expression patterns of genes involved in the biosynthesis of very long chain fatty acids (VLCFA) from *M. oleifera*

KCS, KCR, HCD and ECR proteins sequences in *Arabidopsis* were used as query to search against the protein database of *M. oleifera* using BLASTP program with e-value > 10^−5^. All candidate proteins were further confirmed via SMART/Pfam analysis. To profile their expression in seeds, the transcriptome data generated from the two stages of *M. oleifera* seed development were retrieved from NCBI Sequence Read Archive (SRA) under SRP158484^4^, and mapping onto the *M. oleifera* genome using HISAT2^27^ software. Next, the expression level of each gene was calculated and normalized to fragments per kilobase of transcript per million fragments mapped (FPKM) using STRINGTIE^28^.

## Data Records

The Hi-C sequencing data that were used for the genome assembly have been deposited in the NCBI Sequence Read Archive with accession number SRR18307995^29^ and under BioProject number PRJNA472200. The chromosomal assembly and dataset of gene annotation has been deposited at the Genome Warehouse in National Genomics Data Center, Beijing Institute of Genomics, Chinese Academy of Sciences/China National Center for Bioinformation under accession number GWHBHOG00000000 (https://ngdc.cncb.ac.cn/gwh/Assembly/24427/show) under BioProject accession number PRJCA008620. The genome assembly has also been deposited at DDBJ/ENA/GenBank under the accession JARUNQ000000000^30^. The genome annotations have also been deposited at FigShare^31^.

## Technical Validation

**Fig. 5:**
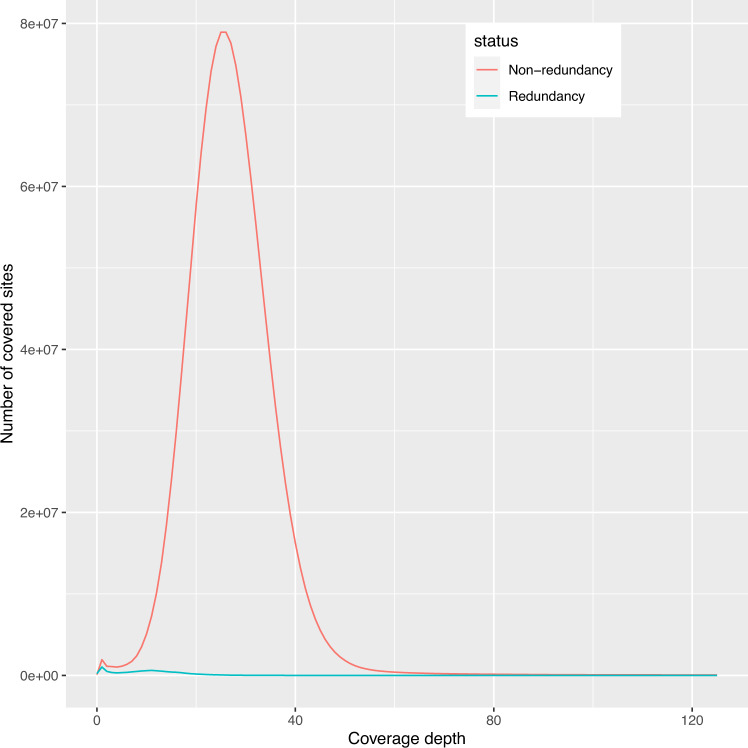
Evaluation of the step of redundancy removal.

**Fig. 6:**
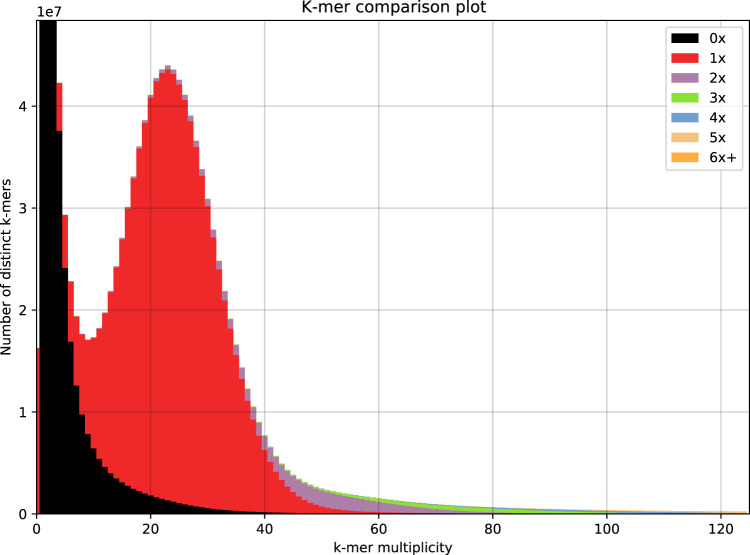
The *k*-mer comparison between final genome assembly and sequencing reads.

The Hi-C heatmap revealed a well-organized interaction contact pattern along the diagonals within/around the chromosome region (Fig. 3), which indirectly confirmed the accuracy of the chromosome assembly. We evaluated the step of redundancy removal and found that redundancy sequences show a coverage depth peak at the half of that of non-redundancy sequences (Fig. 5), indicating they should be true redundancies from duplicated haplotigs. The k-mer comparison plot revealed high consistency between the final genome assembly and sequencing reads (Fig. 6). The heterozygous rate estimated by KAT was 0.12%. The base-level accuracy (QV) and completeness was 34.9 and 95.9%, respectively, as estimated by Merqury. The LAI index was 8.3. The BUSCO completeness of assembly and annotation was 97.5% and 92.2%, respectively.

## Data Availability

All software and pipelines were executed according to the manual and protocols of the published bioinformatics tools. The version and code/parameters of software have been detailed described in Methods.
